# Refractory Bradycardia in the Late Phase of Necrotizing Pancreatitis: A Case Report

**DOI:** 10.7759/cureus.114726

**Published:** 2026-08-18

**Authors:** Youssra Kramchi, Achraf Jeddab, Mouaad Errachki, Marouane Jidal, Si Mohammed Andaloussi, Khalil Mounir, Abdelhamid Jaafari

**Affiliations:** 1 Anesthesiology and Intensive Care Unit, Mohammed V Military Teaching Hospital, Rabat, MAR; 2 Surgical Intensive Care Unit, Mohammed V Military Teaching Hospital, Rabat, MAR

**Keywords:** acute pancreatitis, cardiac arrest, case report, infected pancreatic necrosis, necrotizing pancreatitis, sinus bradycardia

## Abstract

ECG abnormalities are common in acute pancreatitis, yet clinically significant bradyarrhythmias are rarely reported and have been described mainly in the early phase of the disease. We report the case of a 76-year-old hypertensive male who developed severe acute post-endoscopic retrograde cholangiopancreatography pancreatitis, complicated by infected pancreatic necrosis requiring readmission to the intensive care unit on day 20. He was managed with CT-guided drainage and culture-directed antibiotics. In the fourth week of illness, he developed recurrent sinus bradycardia, at times triggered by vagal stimuli (nasogastric tube placement and posture change) and at times occurring spontaneously. On days 25 and 28, he developed severe bradycardia refractory to IV atropine, progressing to cardiac arrest on both occasions; each was managed with brief CPR and a 1 mg IV bolus of epinephrine, with return of spontaneous circulation. On day 30, a further arrest occurred and could not be reversed despite prolonged CPR. Throughout these episodes, serial ECGs showed sinus bradycardia without conduction block, cardiac enzymes and echocardiographic parameters remained unremarkable, electrolytes were within normal range, and inflammatory biomarkers were declining; these findings did not support an alternative reversible cause. We discuss the potential pathophysiological mechanisms underlying this presentation and suggest that life-threatening bradyarrhythmias may also arise in the late phase of necrotizing pancreatitis, raising the case for a low threshold for proactive, aggressive management of bradyarrhythmia in this setting.

## Introduction

Acute pancreatitis (AP) is a frequent cause of transient ECG abnormalities [1]. Although repolarization changes are well documented [2], clinically significant bradyarrhythmias are comparatively rare and have been described in only a limited number of case reports [3]. The pathophysiology of these cardiac manifestations is multifactorial, involving autonomic imbalance, direct myocardial injury, and systemic inflammatory mediators.

Published descriptions of late-phase, recurrent bradycardia in the setting of necrotizing pancreatitis with infected pancreatic collections appear limited. Data on the mortality and outcomes associated with bradyarrhythmias in AP, including its late-phase presentations, are similarly scarce. We report this case to highlight severe bradycardia as a possible late-phase complication of AP and to explore the underlying pathophysiological mechanisms that may precipitate this life-threatening arrhythmia.

## Case presentation

**Figure 1:**
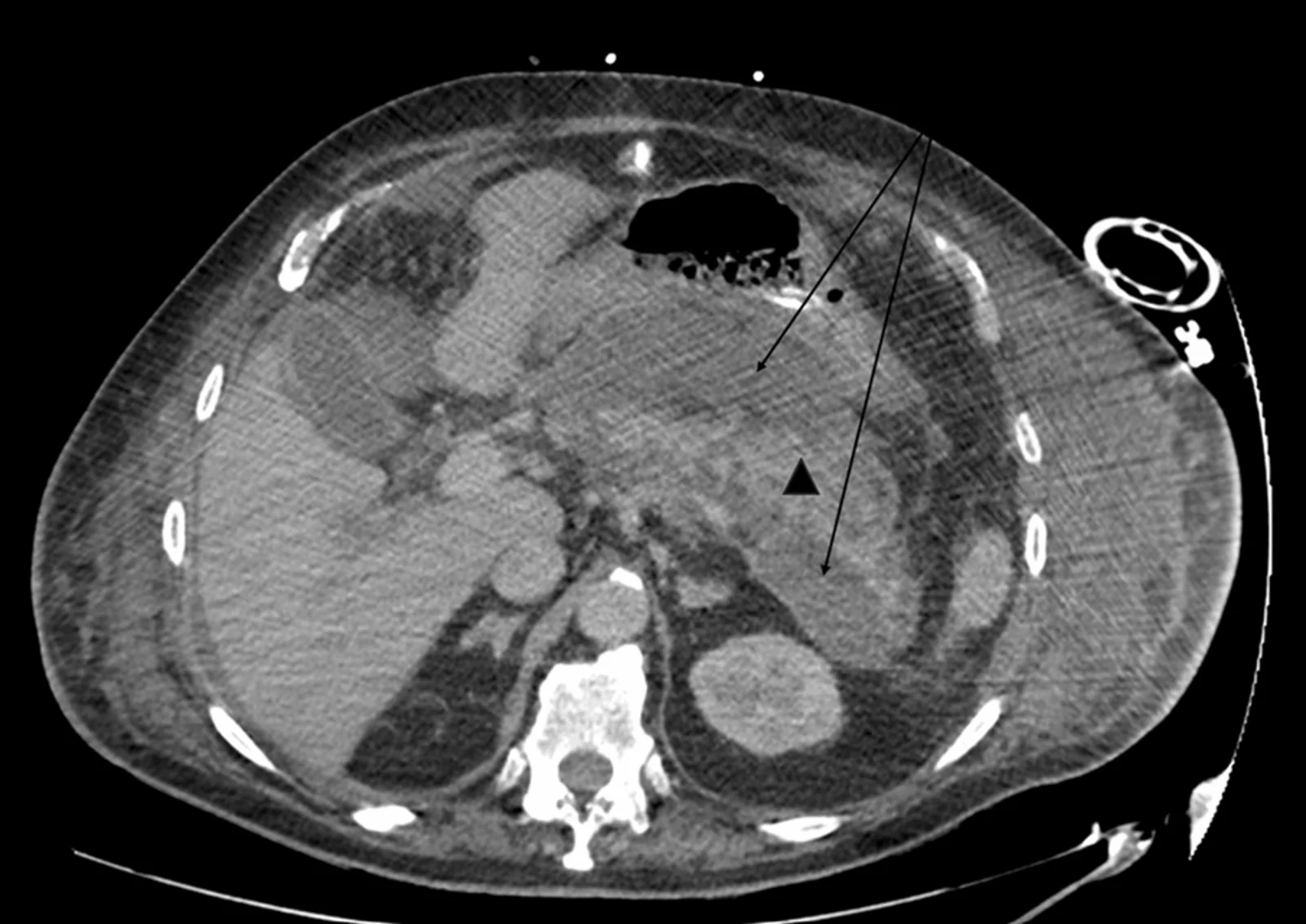
Axial contrast-enhanced abdominal CT scan. The image demonstrates diffuse pancreatic enlargement (arrowhead) with heterogeneous enhancement indicative of necrosis, accompanied by extensive peripancreatic fat stranding and significant fluid collections (arrows) in the peripancreatic and retroperitoneal spaces, consistent with Balthazar stage E classification.

A 76-year-old male with a history of hypertension, previously controlled with amlodipine, underwent endoscopic retrograde cholangiopancreatography for cholangitis and subsequently developed severe AP classified as Balthazar E (Figure 1), complicated by abdominal compartment syndrome, profound hemodynamic instability, acute respiratory failure, severe acute kidney injury, and portal vein thrombosis. By day 18, all early-phase complications had resolved: vasopressor and renal replacement support were no longer needed, intra-abdominal pressure had normalized, and oral feeding was tolerated, permitting transfer to the gastroenterology ward.

**Figure 2:**
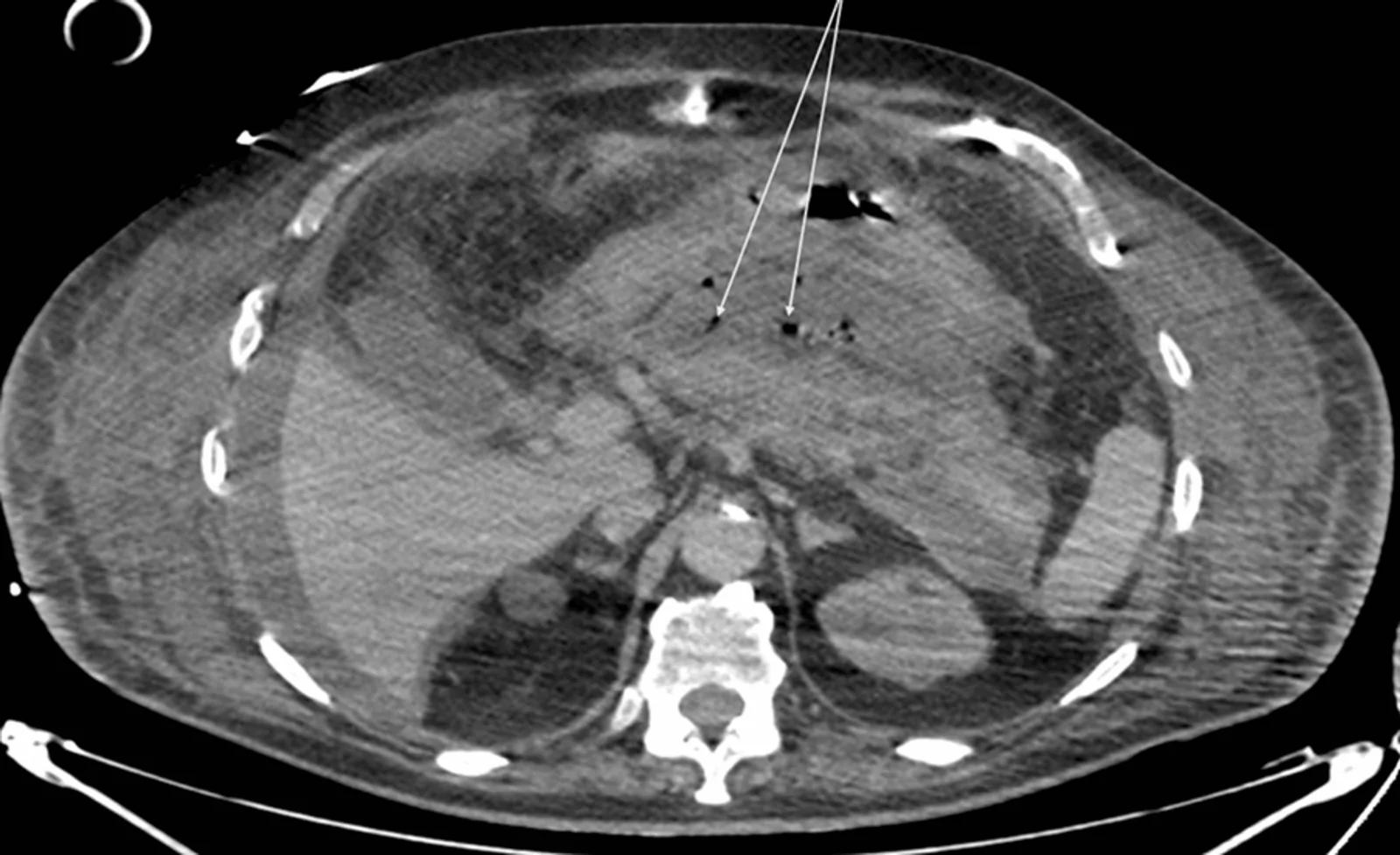
Axial contrast-enhanced abdominal CT scan upon readmission. The image demonstrates the presence of gas bubbles (arrows) within the areas of pancreatic necrosis. This finding is consistent with the diagnosis of infected pancreatic necrosis.

Forty-eight hours later (day 20), he was readmitted for acute respiratory failure requiring intubation and mechanical ventilation. He was maintained on light sedation with fentanyl only, 500-1000 µg/day; no benzodiazepines, propofol, dexmedetomidine, neostigmine, amiodarone, beta-blockers, calcium-channel blockers, or digoxin were administered. Contrast-enhanced CT confirmed infected pancreatic necrosis (Figure 2), managed with CT-guided percutaneous drainage and broad-spectrum antibiotics, later narrowed according to culture results from the drained collection (Table 1). Inflammatory markers (CRP and procalcitonin) followed a steadily decreasing trend throughout this period.

**Table 1:** Chronological clinical timeline. AP, acute pancreatitis; ROSC, return of spontaneous circulation

Time point	Clinical event	Clinical management/response
Day 0	Endoscopic retrograde cholangiopancreatography; onset of severe AP (Balthazar E)	Initial resuscitation
Days 1-18	Multiorgan failure; management of shock and renal insufficiency	Clinical stabilization
Day 18	Transfer to the gastroenterology ward	Patient clinically stable
Days 20-22	Readmission to ICU; infected pancreatic necrosis confirmed	Percutaneous drainage and antibiotics
Day 23	First episode of sinus bradycardia (30-40 bpm)	Spontaneous resolution; no intervention
Days 23-24	Recurrent episodes of sinus bradycardia	IV atropine (0.5-1 mg); initially effective
Day 25	Severe refractory sinus bradycardia leading to cardiac arrest	Atropine ineffective; cardiac arrest, resuscitation, CPR (low-flow 1 min, no-flow 0 min); 1 mg IV epinephrine; ROSC; sinus rhythm
Day 28	Recurrent severe bradycardia; second cardiac arrest	Cardiac arrest, resuscitation, CPR (low-flow 2 min, no-flow 0 min); 1 mg IV epinephrine; ROSC; sinus rhythm
Day 30	Final cardiac arrest	Prolonged CPR (low-flow 40 min, no-flow 0 min); 1 mg IV epinephrine every three to five minutes; no ROSC

**Figure 3:**
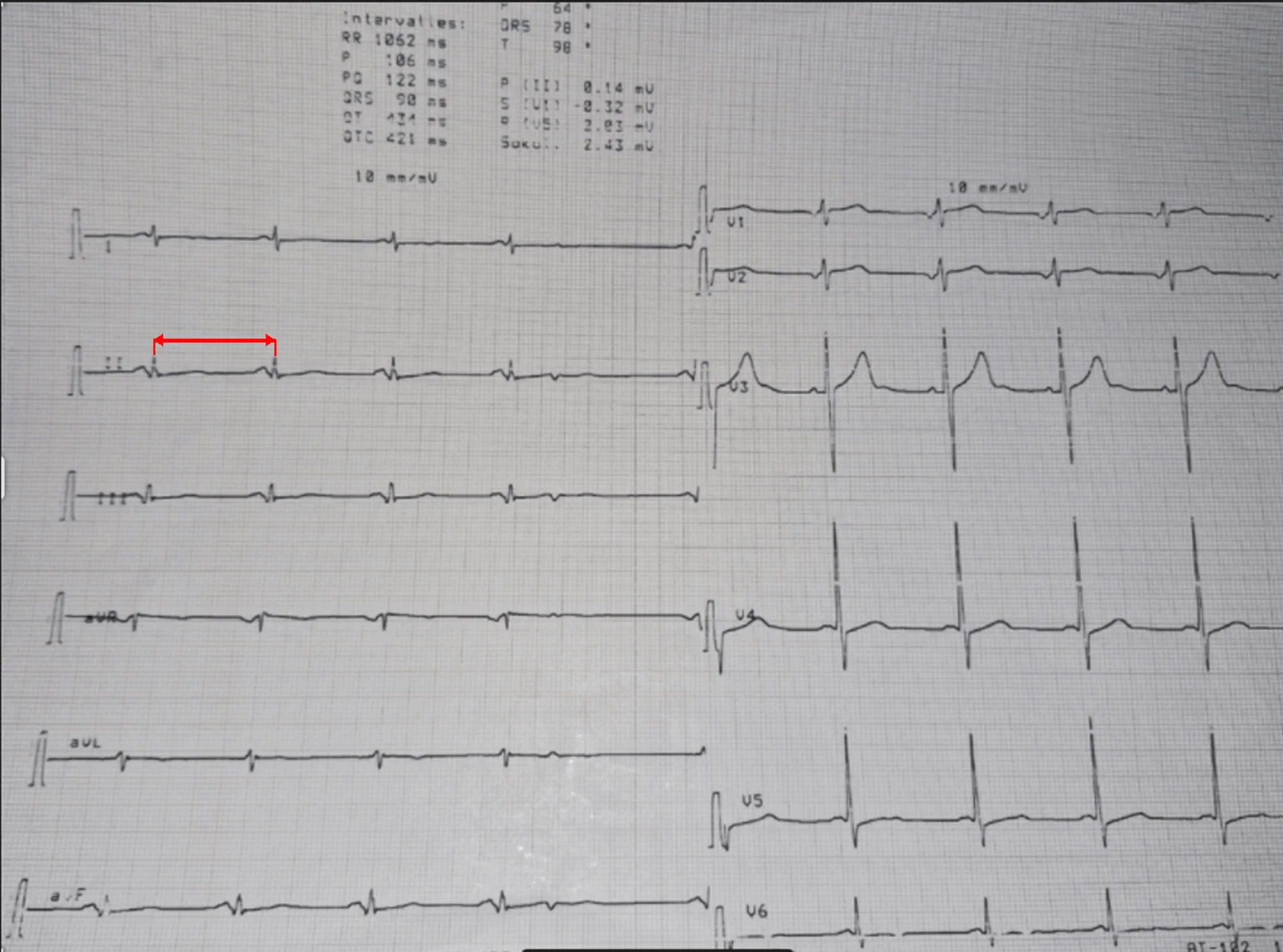
Sinus bradycardia (56 beats/min) on day 23 of AP onset. The arrow indicates the prolonged R-R interval (1062 ms) on lead II. AP, acute pancreatitis

**Figure 4:**
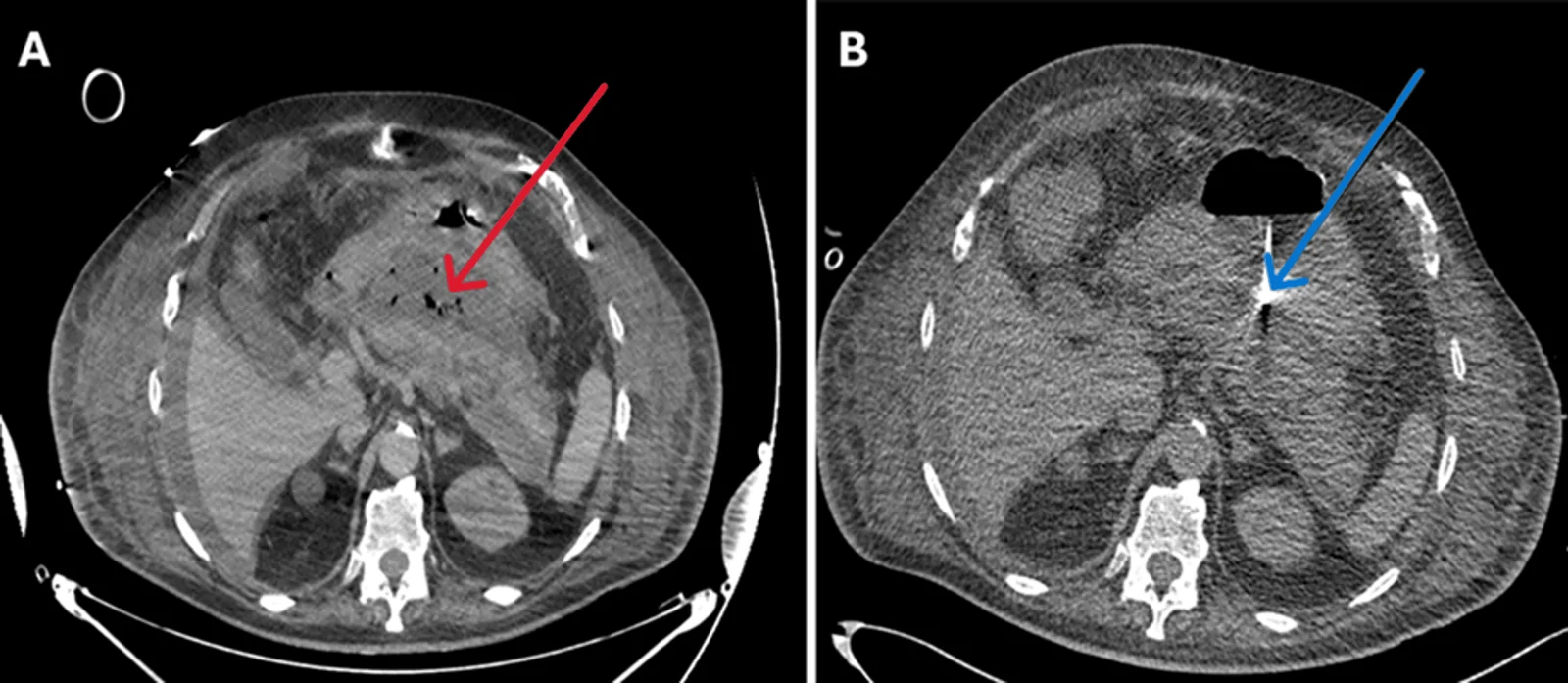
CT assessment of infected pancreatic necrosis before and after percutaneous drainage. (A) Preprocedural axial contrast-enhanced CT scan showing a heterogeneous peripancreatic collection with foci of gas (red arrow), consistent with infected pancreatic necrosis. (B) Postprocedural scan confirming the correct placement of the percutaneous drainage catheter (blue arrow) within the collection, with visible reduction in the collection volume.

On the fourth week of illness (day 23), the patient developed an initial episode of sinus bradycardia (30-40 bpm), which was well tolerated and self-resolving. Two subsequent episodes responded promptly to IV atropine (0.5-1.0 mg). Throughout, the rhythm remained sinus, without conduction block or repolarization abnormalities (Figure 3), and a thorough cardiac and systemic workup found no alternative explanation: troponin levels and transthoracic echocardiography were unremarkable (preserved left ventricular ejection fraction, no wall motion abnormalities, normal right ventricular function, and no pericardial effusion); CT pulmonary angiography excluded pulmonary embolism; electrolyte and acid-base balance were preserved; and the patient was receiving no vasoactive or bradycardia-inducing medications. Intra-abdominal pressure remained below 20 mmHg, and percutaneous drainage of the necrotic collection was effective (Figure 4).

Over the following days, bradycardic episodes became more frequent and prolonged (four to six episodes per day, lasting between two and four minutes), often, though not exclusively, provoked by vagal stimuli such as endotracheal suctioning, nasogastric tube manipulation, or postural change. The response to atropine diminished progressively, and on days 25 and 28, the patient developed severe atropine-resistant bradycardia culminating in cardiac arrest on each occasion; both were rapidly reversed with brief CPR and a single 1 mg IV bolus of epinephrine, with full neurological recovery between episodes. On day 30, a further arrest occurred in the same setting; despite prolonged resuscitation (40 minutes), return of spontaneous circulation could not be achieved.

## Discussion

This case describes a critically ill patient who, during the late phase of severe necrotizing pancreatitis complicated by infected necrosis, developed recurrent sinus bradycardia progressing to atropine-resistant cardiac arrest after a thorough workup excluded the common reversible causes of bradyarrhythmia.

AP is a well-recognized trigger of transient ECG abnormalities, most commonly T-wave flattening and ST-segment depression [1,2,4]. Isolated bradyarrhythmia, however, is reported far less frequently and almost exclusively during the early phase of the disease; late-phase descriptions of AP tend to focus on regional complications, with cardiac involvement receiving comparatively little attention.

Several mechanisms have been proposed to explain bradyarrhythmia in AP, though most were described in the early phase and may not directly translate to our patient’s presentation. The most pertinent to this case is the vagally mediated cardio-biliary reflex [4-7], in which pancreatic inflammation stimulates afferent visceral fibers converging on the nucleus tractus solitarius, producing vagal outflow that slows sinus node automaticity, a mechanism consistent with our patient’s initial responsiveness to atropine and the vagal triggers (endotracheal suctioning, nasogastric manipulation, and positioning) that preceded several episodes. Direct myocardial injury from circulating proteolytic enzymes [4,8], prothrombotic microvascular injury with microthrombi formation within the coronary microcirculation secondary to coagulation cascade activation [1,2,4,9], and electrolyte disturbance are all recognized contributors to arrhythmia in AP more broadly. None of these, however, were supported in our patient: troponin and echocardiography were unremarkable throughout, arguing against significant myocardial or microvascular injury, and electrolytes remained within normal range at the time of the episodes.

A feature distinguishing this case from most reported presentations is the temporal association between bradycardia and late-phase infected pancreatic necrosis, which is itself strongly associated with bacteremia and increased mortality [10]. Circulating bacterial or fungal toxins have been shown experimentally to activate the cholinergic nervous system and increase parasympathetic tone [11], offering a plausible, though unconfirmed, link between ongoing infection and the observed bradycardia in our patient.

The Bezold-Jarisch reflex (BJR), a cardio-inhibitory response triggered by mechanical stimulation of ventricular vagal afferents [12], has been invoked in other settings involving intra-abdominal hypertension and reduced venous return, such as large intra-abdominal masses [12] or pregnancy-related caval compression [13]. In our patient, intra-abdominal pressure remained below 20 mmHg throughout the bradycardic episodes, which does not support significant caval compression as an active mechanism at that time. We therefore raise the BJR only as a hypothesis warranting further investigation in this population, rather than as a likely contributor in this case.

Atropine responsiveness diminished progressively over the course of the illness, a pattern that may reflect several explanations, including evolving nonvagal mechanisms, subclinical conduction system disease, or terminal physiological decline, rather than a specific pharmacological process; the available data do not allow us to distinguish between these possibilities. A comparable case of sinus bradycardia progressing to cardiac arrest in the setting of infected pancreatic necrosis has been previously reported [1], in which a prophylactic regimen of atropine before nociceptive maneuvers was associated with fewer bradycardic episodes. While this observation may be worth considering in similar clinical contexts, the evidence supporting a standardized prophylactic protocol remains limited to isolated reports, and any such approach would need to be evaluated prospectively before being recommended in practice. A comparative summary of published case reports detailing ECG and arrhythmic manifestations in AP is provided in Table 2.

**Table 2:** Published case reports on ECG and arrhythmic manifestations in AP. AP, acute pancreatitis

Reference	Patient (age/sex)	Primary diagnosis	Cardiac manifestation
Challan Belval et al. (2010) [1]	53/M	Severe AP with infected pancreatic necrosis	Recurrent bradycardia, sinus pause, cardiac arrest
Türkay et al. (2009) [2]	47/F	AP	Sinus bradycardia, T-wave inversion (V1-V6)
Denizeau et al. (1976) [3]	N/A	AP	Complete atrioventricular block
Khairy and Marsolais (2001) [4]	64/F	Mild AP	ST elevation, T-wave inversion (mimicking myocardial infarction)
Ro et al. (2004) [9]	43/F	AP	T-wave inversion, anteroapical akinesis
Present case	76/M	Severe AP with infected pancreatic necrosis	Recurrent severe bradycardia, cardiac arrest

As with any single case report, the mechanisms discussed here remain hypothetical rather than proof of causality, and no firm conclusions can be drawn about the relative contribution of each proposed pathway. Nonetheless, this case suggests that severe refractory bradyarrhythmia can occur in the late phase of necrotizing pancreatitis, in addition to its more widely recognized early-phase presentations. Given the rarity and severity of this complication, we suggest that clinicians maintain a lower threshold for continuous cardiac monitoring and early escalation strategies in similar late-phase presentations, while acknowledging that further studies are needed to clarify the underlying mechanisms and to establish evidence-based prevention and management strategies.

## Conclusions

This case illustrates that severe refractory sinus bradycardia can occur during the late phase of necrotizing pancreatitis in a patient with infected pancreatic necrosis after common reversible causes have been excluded. While the underlying mechanisms remain speculative and cannot be established from a single case, this presentation suggests that bradyarrhythmia in AP is not confined to the early phase of the disease. Clinicians managing patients with severe or necrotizing pancreatitis, particularly in the setting of infected necrosis, should maintain awareness of this potential complication and a low threshold for continuous cardiac monitoring, even beyond the initial acute phase. Further studies are needed to clarify the pathophysiology of late-phase bradyarrhythmia in this setting and to guide anticipatory management strategies.
